# A skills network approach to physicians’ competence in shared decision making

**DOI:** 10.1111/hex.13130

**Published:** 2020-09-01

**Authors:** Levente Kriston, Pola Hahlweg, Martin Härter, Isabelle Scholl

**Affiliations:** ^1^ Department of Medical Psychology University Medical Center Hamburg‐Eppendorf Hamburg Germany

**Keywords:** clinical competence, communication, health‐care process assessment, patient participation, patient‐centred care, performance measures, quality improvement, shared decision making

## Abstract

**Background:**

Measurement of physicians’ competence in shared decision making (SDM) remains challenging with frequent disagreement between assessment methods.

**Objective:**

To conceptualize and measure physicians’ SDM competence as an organized network of behavioural skills and to determine whether processing patient‐reported data according to this model can be used to predict observer‐rated competence.

**Design:**

Secondary analysis of an observational study.

**Setting and participants:**

Primary and specialty outpatient care physicians and consecutively recruited adult patients with a chronic condition who faced a treatment decision with multiple acceptable choices.

**Measures:**

Network parameters constructed from patients’ assessment of physicians’ SDM skills as measured by the 9‐item Shared Decision Making Questionnaire (SDM‐Q‐9) and observer‐rated SDM competence of physicians measured by three widely used observer‐rated instruments.

**Results:**

29 physicians (12 female, 17 male; mean age 50.3 years) recruited 310 patients (59.4% female, 40.6% male; mean age 54.0 years) facing a decision mainly regarding type 2 diabetes (36.4%), chronic back pain (32.8%) or depressive disorder (26.8%). Although most investigated skills were interrelated, elicitation of the patient's treatment preferences showed the strongest associations with the other skills. Network parameters of this skill were also decisive in predicting observer‐rated competence. Correlation between predicted competence scores and observer‐rated measurements ranged from 0.710 to 0.785.

**Conclusions:**

Conceptualizing physicians’ SDM competence as a network of interacting skills enables the measurement of observer‐rated competence using patient‐reported data. In addition to theoretical implications for defining and training medical competences, the findings open a new way to measure physicians’ SDM competence under routine conditions.

## 1 INTRODUCTION

The medical interview is a core clinical tool and a major determinant of quality of care, performed up to 300 000 times in the professional lifetime of a practising physician.^1^ Its success depends largely on the physician's competence in shared (or participatory) decision making (SDM), which can be described as a patient‐centred communication process in the medical encounter with the aim of building a consensus about the preferred treatment among multiple acceptable health care choices in accordance with the preferences and values of the patient.^2^, ^3^, ^4^ SDM is mostly characterized by listing core 'concepts' or 'elements', which usually describe communication skills, that is physician behaviours or 'speech actions' that are required for SDM by normative conceptualizations.^5^, ^6^, ^7^ Although definitions of SDM vary, it is largely unequivocal that it includes the justification of deliberative work, the description of the pros and cons of treatment options, information exchange, the elicitation and discussion of patient values and preferences, and the integration of these preferences in the decision.^8^, ^9^, ^10^ In this context, it is meaningful to differentiate between performed behaviour (eg, 'explaining treatment options') and its evaluation (eg, 'explaining treatment options clearly and comprehensively'). The former can be considered to focus on quantification and the latter on qualification of behaviour. Even though this differentiation is context‐dependent and sometimes, particularly in situations without clear evaluation criteria, challenging, we think it is necessary for a deeper understanding of what competence actually is. Here, we refer to specific observable behavioural actions with the term 'skills', while we use 'competence' to label the evaluation of the quality of the performed behaviour.^11^

Measuring complex clinical competences is challenging even under controlled conditions (eg, in training), and it becomes an extremely difficult task in routine practice.^12^, ^13^ Available information sources comprise patient surveys, physician self‐reports, the direct observation of clinical encounters with real or standardized patients, and the rating of audio‐ or videotaped interactions.^14^, ^15^ However, leading experts suggest that both definition and measurement of these competences need to improve.^3^, ^14^

The process of SDM is primarily assessed by patient questionnaires or observer‐rated coding schemes, but the measurement properties of most available tools have been shown to be unsatisfactory or insufficiently tested.^16^ In addition, empirical findings suggest disagreement between patient and observer ratings.^10^, ^16^, ^17^, ^18^, ^19^, ^20^, ^21^, ^22^, ^23^ In the evaluation of these findings, two rarely recognized issues should be noticed. First, a commonly overlooked difference between patient‐rated and observer‐rated tools is that patients are commonly asked to assess whether and to which extent a certain physician behaviour occurred, while observers usually instructed to assess the level of competence of the performed behaviours. Thus, patients assess (rather subjectively) perceived situational skills, whereas observers rate (more objectively) realized competence. Second, even if most measures include the assessment of physician behaviours, their potential to capture physician competence is rarely recognized. Most measures attempt to capture the process of SDM within a unique consultation, while the possible advantages of using information collected on the same physician across several consultations for competence assessment remain rarely exploited.

Recently, modelling latent psychological constructs using networks gained increased attention. In general, networks are systems consisting of components (called 'nodes') that interact with each other through their connections (called 'edges'). Network models showed promising results in several fields, including the investigation of social structures,^24^ mental disorders,^25^ intelligence,^26^ health‐related quality of life,^27^ personality traits,^28^ and attitudes.^29^ The aim of this study was to conceptualize physicians’ SDM competence as an organized network of interacting behavioural skills and to determine whether processing patient‐reported data according to this model can be used to predict observer‐rated competence. In addition, we paid particular attention to documenting the technical details and challenges of our approach carefully in order to provide a methodological framework for similar development and validation efforts.

## 2 METHODS

### 2.1 Design and procedures

From July 2018 to June 2019, we performed secondary analysis of data collected between August 2009 and September 2010 in a cross‐sectional study on measuring SDM in outpatient care in Hamburg, Germany.^17^ We selected primary and specialty care physicians from the Hamburg metropolitan area randomly and invited them to participate with the aim of reaching a sample of thirty physicians. Participating physicians were asked to recruit adult patients with a chronic condition (if possible type 2 diabetes, chronic back pain or depression) who faced a treatment decision with multiple acceptable choices. Each physician agreed to recruit ten patients for filling out a self‐report measure of SDM and to audio‐record consultations with three patients for observer ratings. The planned numbers of documented consultations via self‐report and audio‐recording differed due to the fact that they were collected for partly different primary study aims but were used together in this secondary analysis. The physicians received a financial compensation of 10 Euros per collected patient report measure and 50 Euros for each audio‐recorded consultation. The study protocol of the original study for data collection was approved by the ethics committee of the state chamber of physicians in Hamburg.

### 2.2 Observer‐rated measures of SDM competence

We used assessment by trained observers as a reference criterion of SDM competence, measured by three widely used instruments: the OPTION‐12,^30^, ^31^ the OPTION‐5,^32^, ^33^ and the Invest in the End subscale of the Four Habits Coding Scheme (4HCS).^34^, ^35^ The Invest in the End subscale of the 4HCS does not explicitly aim to measure SDM, but its focus on 'effective decision making and information sharing'^34^ makes it practically identical to SDM. Although none of these instruments name their target constructs 'competence' explicitly, their coding instructions often include descriptors like 'to a good standard',^30^ 'to a high standard',^30^ 'skilled',^32^ 'exemplary',^32^ 'fully',^34^ 'clearly'^34^ and 'effectively',^34^ indicating that an evaluation of the observed behaviours is intended. Although it is not unequivocal which measurement model underlies the OPTION‐12 and the 4HCS, we used them in accordance with a formative measurement model, on which OPTION‐5 is explicitly based.^32^ According to this model, competence in SDM is the average proficiency in the skills that are normatively defined to be the necessary and sufficient components of SDM by the respective authors. In absence of guidance on weighting the specific items, we used the unweighted mean to calculate composite scores for each measure.

All consultations were rated by two of four trained raters (varying across measures) using rating manuals and pilot sessions to achieve sufficient agreement. Raters were blinded to the results of ratings with other measures. For analysis, scores were transformed to range from 0 to 100, with higher values indicating a higher level of competence.

### 2.3 Skills networks of SDM competence

In a typical medical encounter, a series of communication skills can be observed. These skills can be considered nodes of a skills network, of which interactions are represented by the corresponding edges in the network. This interaction can be manifold, and, basically, it may refer to any kind of association between skills, such as simple temporal ordering, co‐occurrence due to constraints by the context, logically given hierarchical relations, strict causal connections or any mixture of these. Frequently, the connections will be rhetorical relations, such as elaboration, justification, conditioning and evaluation.^36^ For example, informing on the pros and cons of treatment options is often precluded by listing the available treatment options, leading to an association of these two skills. In this framework, we define competence as a systematic way of using skills in the encounter, that is as a specific structure of the nodes and the edges in the skills network exposed across consultations. Hereby, we explicitly acknowledge that in any encounter, SDM skills are not necessarily shown in a predefined normative order but are rather organized in a complex dynamic process with iterative, recursive and non‐linear features.^6^, ^7^, ^37^, ^38^

Participating patients filled out the 9‐item Shared Decision Making Questionnaire (SDM‐Q‐9) directly after the consultation with their physician.^39^ This measure asks patients to rate the extent certain behaviours were shown by the physician in the consultation using a six‐step Likert‐type scale ranging from zero to five. By rating the extent to which a behaviour occurred but not appraising how well it was performed, the tool measures rather skills than competence. Each item of the SDM‐Q‐9 corresponds to an SDM skill (Table 1).

**TABLE 1:** Skills measured by the items of the 9‐item Shared Decision Making Questionnaire (SDM‐Q‐9)

Item	Label	Description
1	Focusing the decision	Making clear to the patient that a decision needs to be made
2	Sharing the decision	Asking the patient to which extent he or she wishes to participate in the decision‐making process
3	Presenting options	Informing the patient that different treatment options exist for his or her condition
4	Informing on options	Explaining the pros and cons of the treatment options to the patient
5	Supporting comprehension	Supporting the patient in comprehending all information
6	Eliciting preferences	Asking the patient about his or her preferred treatment option
7	Deliberating the decision	Deliberating the decision together with the patient
8	Selecting an option	Selecting a treatment option together with the patient
9	Planning actions	Reaching an agreement with the patient on how to proceed

### 2.4 Statistical analysis

For observer‐rated measures, mean scores across consultations were calculated for each physician as a measure of their SDM competence (Box 1). Multilevel variance decomposition was performed to calculate intracluster correlation coefficients. Inter‐rater reliability was estimated with intraclass correlation coefficients calculated with fixed and random rater effects from three‐level models that were generalized from standard formulas.^40^

BOX 1 **Assessing Competence from Observer‐Rated Competence Data**

1 Consider an observer‐rated measure with 10 items (i1 to i10), each measuring competence in a specific skill, used by two independent raters (r1 and r2) to rate 5 independent patient consultations (c1 to c5) of each of 30 physicians (p1 to p30), respectively.

*Obtain single‐rated single‐consultation competence*

1. Calculate the composite score (usually sum or average) of the items i1 to i10 for consultation c1 of physician p1, rated by rater r1.
2. Repeat step 1 for rater r2.

*Obtain multiple‐rated single‐consultation competence*

- Calculate the average of the values obtained in step 1 and step 2.
- Repeat steps 1 to 3 for consultation c2 to c5 of physician p1.

*Obtain multiple‐rated multiple‐consultation (ie, physician‐level) competence*

- Calculate the average of the values obtained in step 4.
- Repeat steps 1 to 5 for physician p2 to p30.

*Notes*

- Consider using scores transformed to a scale from 0 to 100 instead of raw scores (done in this study).
- Considering checking the quality of the measure by calculating multilevel variance decomposition and assessing inter‐rater reliability on both the patient and the physician levels (done in this study).
- Consider using multilevel modelling for estimating competence scores in order to use the total amount of available information and enhance precision (not done in this study).

Based on the patients' self‐reports (SDM‐Q‐9), a directed and weighted network with skills as nodes was estimated for each physician using a multilevel framework (Box 2). Our approach to estimating the skills network was inspired by the multilevel vector autoregression methodology proposed by Bringmann and colleagues^41^; however, we used cross‐sectional instead of longitudinal data for creating the network estimates. In the network, the direction of an edge between two nodes describes the way of impact of one node on another, while its weight denotes the strength of this impact. For calculating the edges, each skill of the SDM‐Q‐9 was regressed on all other skills in a multilevel regression model while allowing the intercept and all slopes vary across physicians in some models and fixing them in others. In random slopes models, regression coefficients describing the directed association of each skill with each other skill were obtained for each physician. These models used all available data and borrowed strength for estimates for a certain physician from data of all other physicians. In order to exclude possibly spurious associations, each network was purged by removing edges with weights that were not statistically significantly higher than zero. The remaining coefficients were treated as network weights and used for the calculation of the node parameters.

BOX 2 **Assessing Competence from Patient‐Rated Skills Data**

1 Consider a patient‐rated measure with 8 items (i1 to i8), each measuring intensity or frequency of a specific skill, used in 15 independent patient consultations (c1 to c15) of each of 20 physicians (p1 to p20), respectively.

*Create a skills network*

1. Use multilevel regression with the intercept and slopes varying across physicians to estimate the multivariable association of item i1 (outcome) with all other items i2 to i8 (predictors).
2. Repeat step 1 with items i2 to i8 as outcomes.

*Calculate network parameters*

- Calculate activation of skill i1 for physician p1 as the intercept for physician p1 obtained in step 1.
- Calculate outstrength of skill i1 for physician p1 as the sum of the regression coefficients for physician p1 obtained in step 2 with item i1 as predictor.
- Calculate instrength of skill i1 for physician p1 as the sum of the regression coefficients for physician p1 obtained in step 1 with item i1 as outcome.
- Repeat steps 3 to 5 for skills i2 to i8.
- Repeat steps 3 to 6 for physicians p2 to p20.

*Calculate competence estimate*

- Estimate the level of competence for physician p1 using a weighted sum of the network parameters obtained in steps 3 to 6, with the weights obtained from research predicting observer‐rated competence from patient‐rated skills networks.
- Repeat 8 for physician p2 to p20.

*Notes*

- Consider purging/regularizing regression coefficients while creating a skills network in order to avoid processing spurious associations (done in this study).
- Consider using Bayesian analysis in case of limited amount of data (done in this study).
- Consider using non‐directed associations in the skills network in order to simplify the analyses (not done in this study).
- Consider creating the skills network for each physician in a separate analysis in order to make the estimation independent of data from other physicians (only feasible if a large amount of data is available for each physician, not done in this study).

We estimated three widely used node parameters: (a) average skill activation, calculated as the mean of the respective item across consultations, which can be interpreted as the frequency and intensity of showing the skill; (b) skill outstrength, calculated as the sum of the weighted edges outgoing from a certain node, which can be interpreted as the degree of influence of the skill on the other skills; and (c) skill instrength, calculated as the sum of the weighted edges heading towards a certain node, which can be interpreted as the degree to which the skill is influenced by the other skills.^42^ Outstrength and instrength were calculated using the igraph software package.^43^ In network plots, nodes were placed using the force‐directed layout algorithm by Fruchterman and Reingold, with the distance between nodes indicating the strength of the association between them.^44^

For investigating the association of skills network parameters with observer‐rated levels of competence, we performed a series of regression analyses. First, activation, outstrength and instrength of the skills were used as predictors in three separate models for each observer‐rated outcome, respectively. Only skills for which at least one node parameter showed a very likely (95 per cent) non‐zero association with at least one observer‐rated measure were retained in the final models. Predictors were grand‐mean centred in all analyses.

In order to avoid possibly biased estimates obtained from fitting complex models in a relatively small sample, all analyses were performed in a Bayesian framework.^45^, ^46^, ^47^ For Bayesian estimation, we used Markov chain Monte Carlo sampling methods in WinBugs 1.4.3.^48^ Parameter estimates were given uninformative priors and are reported with 95% credible intervals. Results were obtained from 90 000 iterations with a thinning rate of 30 after dropping 60 000 burn‐in simulations. We ran three independent Markov chains with different starting values and checked convergence visually and with Gelman and Rubin's convergence diagnostic.^49^ We used the deviance information criterion for comparing models with a difference of three or more points considered relevant.^50^ Estimates are reported with 95 per cent credible intervals describing the range of values within which a parameter falls with a 95 per cent probability (roughly corresponding to confidence intervals in frequentist statistics). Aligning with traditional frequentist statistical interpretation, we label parameter estimates that exclude zero with 95% probability as 'statistically significant'.

### 2.5 Patient and public involvement and engagement

Patients were not included in planning, conducting or reporting of this secondary analysis.

## 3 RESULTS

### 3.1 Sample

A total of 33 physicians were included in the original study, of which data were used here. The most frequent reported reasons to decline participation were lack of time, lack of interest and anticipated patient recruitment problems.^17^ We included 29 physicians (of whom 24 provided audio‐recordings) and 310 patients (of whom 80 participated in audio‐recorded consultations) with sufficient data in the present analysis. Some physicians required substantial support from the study team for patient recruitment. Availability of data across measures is detailed in Supplementary Table 1.

The mean age of the participating physicians (12 female, 17 male) was 50.3 years (range 35 to 66 years). The majority (21/29) had a specialty in family or in internal medicine. The mean of years of experience was 12.6 (range 1 to 33 years). The subsample of physicians providing at least one audio‐recording did not differ substantially from the total sample (Supplementary Table 2).

The recruited patients (59.4 per cent female, 40.6 per cent male) had a mean age of 54.0 years (range from 18 to 93 years), covering various levels of formal education. Most patients were of German mother tongue and mainly faced a decision regarding type 2 diabetes, chronic back pain or depressive disorder. The subsample of patients of whom consultations were audio‐recorded was comparable to the total sample with a slight underrepresentation of patients with a high formal education and of a mother tongue other than German (Supplementary Table 3).

### 3.2 Observer‐rated measures of SDM competence

Multilevel variance decomposition showed intracluster correlations between 0.27 and 0.54, indicating that the measures captured a considerable amount of between‐physician variance (Supplementary Tables 4 and 5). Inter‐rater reliability ranged from 0.69 to 0.76 across instruments for the physician level, showing an acceptable agreement between raters (Supplementary Table 6). On a scale from 0 to 100, mean scores across all physicians were 16.15, 11.83 and 33.16 for the OPTION‐12, the OPTION‐5 and the Invest in the End subscale of the 4HCS, respectively. Physician‐level correlation between the three observer‐rated measures ranged from 0.50 to 0.75, suggesting largely overlapping but not fully identical constructs (Supplementary Tables 4 and 5).

### 3.3 Average skills network of SDM competence

The skill‐wise analyses for estimating the edges of the skills network suggested that for all but one skill (focusing the decision, skill 1 of the SDM‐Q‐9), models with a random intercept and random slopes fit the data better than models with fixed parameters (Supplementary Table 7). This implies that physicians differ statistically significantly regarding the activation and interrelation of the investigated skills.

**FIGURE 1:**
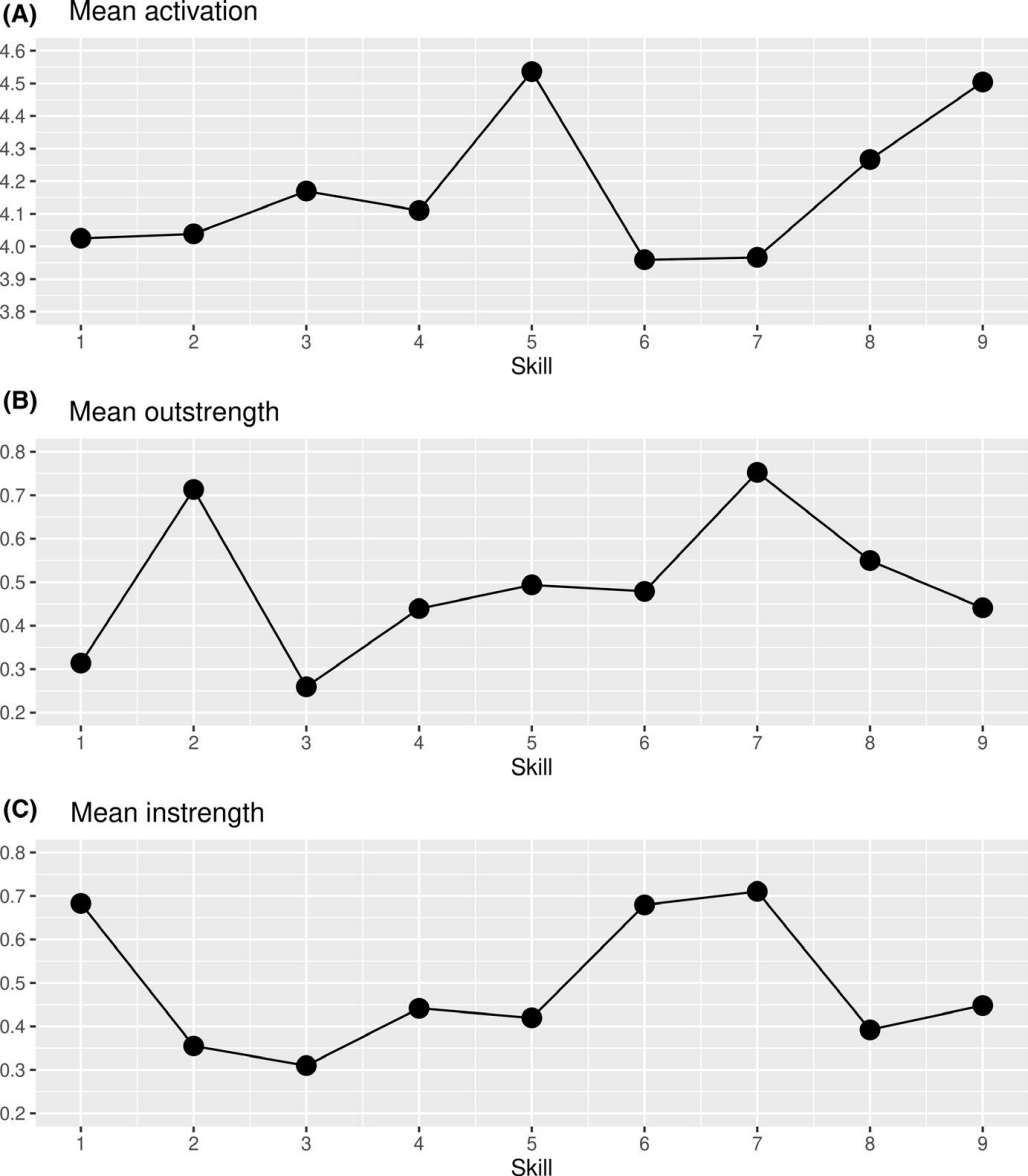
Node parameters of each skill in the average skills network of SDM competence. Skills refer to the items of the 9‐item Shared Decision Making Questionnaire (SDM‐Q‐9), cf. Table 1

At average, supporting comprehension (skill 5) and planning actions (skill 9) were the most frequently and intensively shown skills (Figure 1, panel A). Sharing the decision (skill 2) and deliberating the decision (skill 7) showed the strongest influence on other SDM skills (Figure 1, panel B). Focusing the decision (skill 1), eliciting preferences (skill 6) and deliberating the decision (skill 7) were most strongly influenced by other skills (Figure 1, panel C).

**FIGURE 2:**
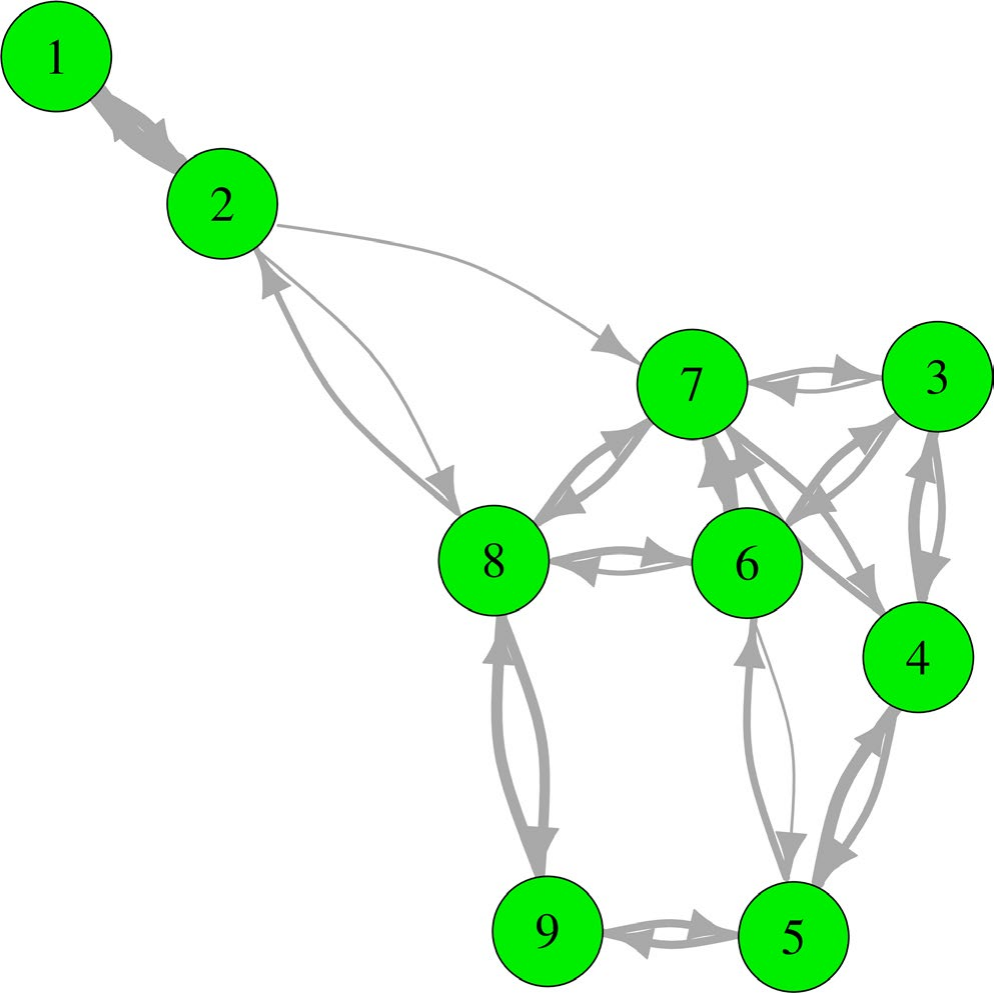
Average skills network of SDM competence. Numbers refer to the skills of the 9‐item Shared Decision Making Questionnaire (SDM‐Q‐9), cf. Table 1. Arrow size is proportional to the strength association between skills

The network plot of the skills reveals several mutual associations between skills (Figure 2). Eliciting preferences (skill 6) seems to build the centre of this process, forming a dense and strongly connected block with deliberating the decision (skill 7), presenting options (skill 3), informing on options (skill 4) and selecting an option (skill 8). Somewhat less intensely but still substantially related to this core are the skills of supporting comprehension (skill 5) and planning actions (skill 9). The strongly interrelated pair of focusing the decision (skill 1) and sharing the decision (skill 2) is only moderately connected to the other skills. The skills network of each physician is depicted in the Supplementary Figure 1.

### 3.4 Predicting observer‐rated SDM competence from skills networks

**FIGURE 3:**
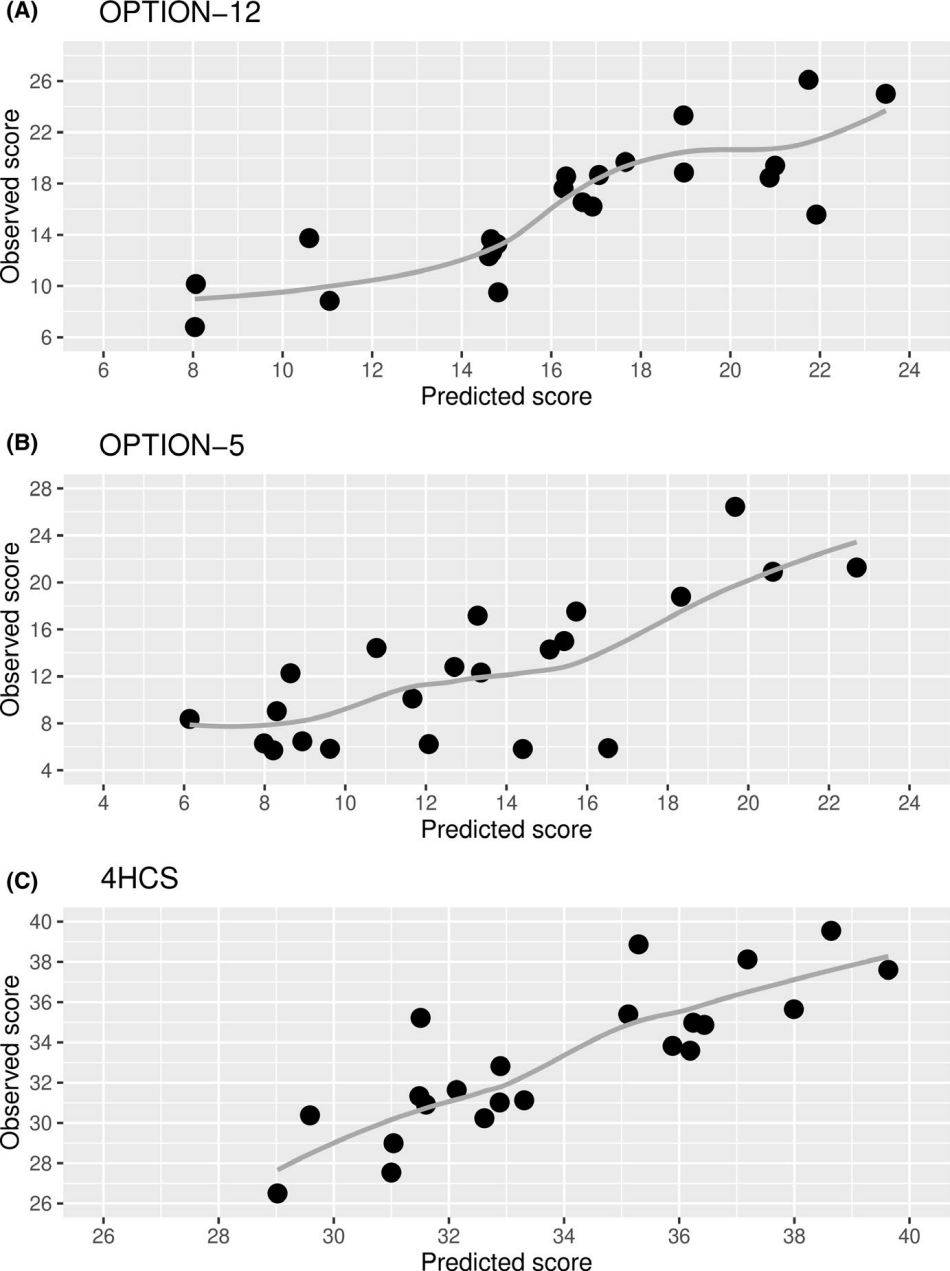
Scatterplot of observed and predicted values of SDM competence. Points correspond to physicians. Grey lines are locally estimated scatterplot smoothing (loess) curves. 4HCS, Invest in the End subscale of the Four Habits Coding Scheme

Activation of focusing the decision (skill 1), outstrength and instrength of eliciting preferences (skill 6) and outstrength of deliberating the decision (skill 7) were associated with at least one of the three observer‐rated measures of SDM competence in parameter‐specific analyses (Supplementary Tables 8 to 10). Therefore, the final model retained activation, outstrength and instrength of these skills as potential predictors of the three observer‐rated measures of SDM competence. As shown in Table 2, eliciting preference (skill 6) played a significant role in most models. Its activation was positively associated with SDM competence as measured by the OPTION‐12 and the Invest in the End subscale of the 4HCS, its outstrength with the Invest in the End subscale of the 4HCS and its instrength with the OPTION‐5 measure. In addition, the outstrength of deliberating the decision (skill 7) was negatively associated with OPTION‐12 scores. It should be noted that correlation between parameters ranged roughly from −0.70 to 0.70, which complicates direct interpretation of single estimates due to possible multicollinearity issues. The models accounted for more than half of the variance in each outcome, showing strong association between predicted and observed values ranging between 0.70 and 0.80 across measures. Model‐based predictions and observed values are contrasted in Figure 3.

**TABLE 2:** Multiple linear regression model weights to predict observer‐rated measures of shared decision‐making competence from node parameters of selected skills of the SDM‐Q‐9

	OPTION‐12	OPTION‐5	4 HCS
Intercept	16.15 (14.47 to 17.85)	12.44 (10.02 to 14.88)	33.20 (32.05 to 34.36)
Mean			
Skill 1	35.66 (−50.54 to 120.11)	−31.30 (−155.40 to 90.33)	−34.35 (−93.45 to 23.56)
Skill 6	62.74 (11.82 to 114.31)^b^	54.99 (−18.35 to 129.21)	36.71 (1.81 to 72.05)^b^
Skill 7	1.02 (−42.20 to 45.34)	14.67 (−47.55 to 78.51)	27.77 (−1.86 to 58.15)
Outstrength			
Skill 1	−7.11 (−17.30 to 3.10)	−6.02 (−20.69 to 8.68)	−4.55 (−11.54 to 2.44)
Skill 6	9.48 (−1.12 to 19.98)	−0.17 (−15.44 to 14.96)	11.75 (4.49 to 18.95)^b^
Skill 7	−11.21 (−19.00 to −3.13)^b^	−10.46 (−21.68 to 1.17)	−3.62 (−8.96 to 1.91)
Instrength			
Skill 1	13.75 (−47.57 to 72.51)	17.21 (−71.12 to 101.82)	13.25 (−28.73 to 53.52)
Skill 6	5.49 (−4.81 to 15.57)	15.05 (0.20 to 29.58)^b^	3.81 (−3.25 to 10.72)
Skill 7	−1.50 (−12.09 to 8.76)	−0.61 (−15.87 to 14.18)	−4.27 (−11.53 to 2.76)
Residual variance	16.25 (6.78 to 36.00)	33.74 (14.08 to 74.73)	7.63 (3.18 to 16.91)
Model variance	25.11 (11.62 to 45.80)	34.81 (13.26 to 69.57)	12.32 (5.53 to 21.91)
*R*	0.778 (0.603 to 0.885)	0.710 (0.518 to 0.838)	0.785 (0.610 to 0.888)
*R* ^2^	0.611 (0.363 to 0.783)	0.510 (0.268 to 0.702)	0.621 (0.372 to 0.788)
Adjusted *R* ^2^	0.320 (−0.114 to 0.619)	0.143 (−0.280 to 0.478)	0.337 (−0.100 to 0.629)
Residuals^a^	22.0	22.0	22.0

Note

Skills refer to the items of the 9‐item Shared Decision Making Questionnaire (SDM‐Q‐9), cf. Table 1.

All estimated parameters reported with 95% credible interval.

^a^ Compare to 22 data points

^b^ Regression slope parameter is different from zero with a probability of at least 95%

## 4 DISCUSSION

In the present study, we outlined an approach to conceptualization and measurement of SDM competence based on network theory. The proposed theoretical model stresses that definitions of complex patient‐centred communication competences should consider the interrelations and pattern of observable physician behaviours rather than treating them in isolation.

Authors of most existing measures of different aspects of SDM would probably agree that the captured behaviours, experiences or aspects are likely to be interrelated. However, these associations are only addressed in measures with a reflective measurement model, but in most cases even there rather implicitly and without sufficiently defining what the latent constructs behind the observed indicators actually are. More often, associations between items remain unaddressed both conceptually and for the calculation of a composite score. In our approach, we highlight the possibility that the strength of association between SDM‐related skills (behaviours) can be informative of SDM competence. For example, a behaviour that is strongly correlated with other behaviours can be expected to be shown, when other behaviours are shown, but not, when others are not; that is, its occurrence is harmonized with those of the other behaviours. We postulate that these associations can be both conceptually meaningful and used for quantitative assessment of SDM competence. By doing so, we consider the interdependences between skills as a sign of organization of behaviour and presume that difference between various levels of competence is reflected by the attributes of this organization. For estimating SDM competence, it means that the strength of the correlation among behaviours is explicitly used (as outstrength and instrength parameters of the skills networks) to estimate competence. To our knowledge, none of the attempts to measure SDM has utilized this information so far. We have outlined a particular way of quantitative measurement (several others are conceivable) and showed that patient ratings of physician skills can be used to predict physicians’ SDM competence, possibly even with a high accuracy.

It is important to note that we did not aim to provide a complete account of SDM processes in the medical encounter. We focused on the definition and measurement of physicians’ SDM competence based on patient‐rated data concerning physicians’ skills. Within this context, we neither intend to imply that patients do not contribute to SDM processes nor suggest that our approach invalidates SDM frameworks with more emphasis on patient behaviour and experience. We are convinced that SDM is a multifaceted construct, and here, we were interested in what the physician can do to foster SDM, that is the competence a physician needs to make SDM happen in any encounter. An interesting consequence of focusing on the physician level of practice variance is that it was not necessary to collect perfectly paired self‐rated and observer‐rated data on the same consultations; the patient subsamples providing self‐report and participating in audiotaped consultations were not and did not need to be identical. Although differences between patients with self‐report and audiotaped consultations were marginal in our sample, we cannot exclude that the patients were differentially selected for self‐report and for audiotaped consultations by the participating physicians. Somewhat paradoxically, however, a differential selection would even strengthen the claims of the study, because an empirical association between competence based on patient ratings and competence based on observer‐rating were found, even though the data bases comprised mostly different consultations.

Our findings suggest that elicitation of patient preferences might be the core skill in SDM, both directly triggering other behaviours and being their objective. This skill has emerged as the most central one in the average SDM skills network, and its role (activation, outstrength, instrength) proved to be decisive in predicting the results of observer‐rated measures. Also others consider preference elicitation to be 'at the heart of shard decision making'^8^ ^(p267)^ or describe operationalizing SDM techniques in practice as 'reorienting complex treatment and management decisions around patients’ preferences'.^51^ ^(p28)^ Nevertheless, this does not mean that it is sufficient to focus on this skill and its measurement. Its outstrength and instrength are defined by the edges that connect it with the other nodes, making it necessary to obtain information on all skills represented in the network.

Shared decision making is continuously gaining importance in quality improvement^3^, ^4^, ^10^, ^52^ and value‐based care initiatives.^53^, ^54^, ^55^ If confirmed, the presented findings offer a way of assessing SDM competence of physicians with a truly patient‐reported experience measure, which could be readily implementable under routine care conditions with minimal efforts and avoiding tedious external observation. As the calculations require input from several independent patients and simply scoring high on single skills does not necessarily result in the highest competence estimate, manipulation becomes extremely difficult. In consequence, the network framework provides a realistic possibility of continuous evaluation and monitoring of SDM competence both in education and training and in routine practice. The generalization of the approach to teams, institutions, geographic regions or administrative units is also conceivable.

Our findings should be interpreted in a strictly explorative manner and in the light of several limitations. First, the results from a possibly self‐selected sample of physicians treating patients with chronic conditions in a German metropolitan area might not be generalizable to other settings.^56^ Even though findings were similar across three established observer‐rated measures, we cannot confidently judge on stability and generalizability of our conclusions, and thus, investigations in other settings should be carried out. In addition, the physicians’ average level of SDM competence in our sample was somewhat lower than the level of competence in comparable samples as measured by the OPTION‐12,^57^ OPTION‐5^58^ and the 4HCS.^34^ Even though no consensual thresholds for interpretation of these measures exist, the descriptive results of the presented study imply that our approach could not be tested across the full range of SDM competence. Second, most of our estimates were statistically imprecise, leaving room for uncertainty. Due to collinearity among network parameters, limited variance in the observer‐rated competence of the participating physicians, and substantial model complexity relative to the sample size, individual estimates should be considered preliminary approximations and interpreted, if at all, with extreme care. Third, our model‐building approach for predicting scores of observer‐rated measures form skills networks, though set a priori, represents only one of several possibilities. Another way of combining and selecting regression models may lead to other outcomes. Fourth, due to the small sample, our results could not be cross‐validated via data partitioning. However, the use of a Bayesian approach might have reduced this problem to some degree by focusing on probabilistic statements rather than on multiple significance testing.

Several of these limitations could be addressed in larger studies. We consider our sample of roughly 30 physicians, 10 patients rating each physician's SDM skills, 3 audiotaped consultations per physician and 2 independent observers per consultation as the lower limit for performing similar investigations. Based on the computational process of estimating skills networks from patient ratings as well as multilevel variance estimates and inter‐rater reliability of the observer‐rated measures (see Boxes 1 and 2), it seems that a well‐designed study with similar aims should include at least 30 (better 50) physicians, around 30 patients rating SDM skills per physician, a minimum of 5 (better 7) audiotaped consultations per physician and 3 trained observers per consultation. This is fairly in line with suggestions for research on clinical competence^13^ and would also be sufficient for using standard maximum‐likelihood estimation methods.^59^ Substantially smaller studies should preferably use Bayesian estimation with informative priors that can be derived from the results of this investigation.^60^

In addition to testing the predictive part of the presented model, the theoretical side of our approach also needs further attention. Our hypothesis stating that the definitions of competence as a composite of proficiency in specific skills (here, using observer‐rated data) and as a particular pattern of organizing behavioural skills (here, using a network model of patient‐rated data) may be functionally equivalent poses an exciting topic for future work. Further conceptual investigations should focus on elucidating the nature of relations between the skills, the modelling of the skills together with skills belonging to similar competences (eg, building and maintaining a therapeutic relationship) and the investigation of the dependency of the skills network on context factors.^14^, ^61^, ^62^, ^63^ Understanding how various skills and competences interact with each other could provide a unique opportunity to analyse physician behaviour and to give precise advice for improvement in form of highly personalized feedback. Furthermore, simultaneous modelling of physician and patient behaviours in interaction could shed a new light on interpersonal processes.

## 5 CONCLUSIONS

Although still at the stage of a working hypothesis, the skills network model of SDM competence is both conceptually and empirically promising. Being able to investigate, evaluate and train complex clinical competences in a theoretically anchored, empirically validated and technically feasible manner has implications that reach far beyond SDM. Considering clinical competences as dynamically organized systems of skills can enrich and deepen our understanding of the medical profession as a whole.

## CONFLICT OF INTEREST

LK, MH and IS report academic, but not financial, conflict of interest as the developers of the utilized self‐report measure, the SDM‐Q‐9.

## Supporting information

Supplementary Material

## Data Availability

Deidentified individual participant data that support the results will be shared 12 to 36 months following publication. Investigators who propose to use the data have to provide a methodologically sound proposal directed to the corresponding author. Signing a data use/sharing agreement will be necessary, and data security regulations both in Germany and in the country of the investigator who proposes to use the data must be complied with. Support by the study authors depends on available resources.
