# Supplementary material for: A skills network approach to physicians’ competence in shared decision making
Source: Health Expect. 2020 Sep 1;23(6):1466–76. doi: 10.1111/hex.13130 (PMC7752189; doi:10.1111/hex.13130)

Supplementary Online Content

Kriston L, Hahlweg P, Härter M, Scholl I. A skills network approach to physicians' competence in shared decision making.

Supplementary Table 1. Data structure
Supplementary Table 2. Characteristics of participating physicians
Supplementary Table 3. Characteristics of participating patients
Supplementary Table 4. Multilevel variance decomposition and association analyses of the observer measures in available cases
Supplementary Table 5. Multilevel variance decomposition and association analyses of the observer measures in complete cases
Supplementary Table 6. Inter-rater reliability of observer measures
Supplementary Table 7. Model fit of the regression models used to create skills networks
Supplementary Table 8. Multiple linear regression model weights to predict observer-rated measures of shared decision making competence from activation of the skills of the SDM-Q-9
Supplementary Table 9. Multiple linear regression model weights to predict observer-rated measures of shared decision making competence from outstrength of the skills of the SDM-Q-9
Supplementary Table 10. Multiple linear regression model weights to predict observer-rated measures of shared decision making competence from instrength of the skills of the SDM-Q-9
Supplementary Figure 1. Skills network plots of the participating physicians

Supplementary Table 1. Data structure

	Available data						
	**SDM-Q-9**	**OPTION-12**	**OPTION-5**	**4 HCS**	**Number of patients**	**Number of physicians**	**Mean patients/ physicians**
	yes	no	no	no	230	29	7.93
	yes	yes	yes	yes	60	21	2.86
	yes	no	no	yes	7	4	1.75
	no	yes	yes	yes	6	6	1.00
	yes	yes	yes	no	3	3	1.00
	no	no	yes	no	2	2	1.00
	yes	yes	no	no	1	1	1.00
	no	yes	yes	no	1	1	1.00
Number of patients	301	71	79	66	310	29	10.69
Number of physicians	29	22	24	22	29		
Mean patients/ physicians	10.38	3.23	3.29	3.00	10.69		

Supplementary Table 2. Characteristics of participating physicians

	Total sample (n=29)		Sample providing audio recording (n=24)	
	**n**	**(per cent)**	**n**	**(per cent)**
Sex				
female	12/29	(41.4)	11/24	(45.8)
male	17/29	(58.6)	13/24	(54.2)
Age				
30 to 39 years	2/29	(6.9)	2/24	(8.3)
40 to 49 years	14/29	(48.3)	13/24	(54.2)
50 to 59 years	6/29	(20.7)	4/24	(16.7)
> 60 years	7/29	(24.1)	5/24	(20.8)
Specialty				
family medicine	12/29	(41.4)	11/24	(45.8)
internal medicine	9/29	(31.0)	8/24	(33.3)
orthopedics	4/29	(13.8)	3/24	(12.5)
psychiatry	4/29	(13.8)	2/24	(8.3)
Experience				
< 10 years	11/29	(37.9)	11/24	(45.8)
10 to 19 years	12/29	(41.4)	9/24	(37.5)
20 to 29 years	4/29	(13.8)	2/24	(8.3)
30 to 39 years	2/29	(6.9)	2/24	(8.3)

Supplementary Table 3. Characteristics of participating patients

	Total sample (n=310)		Sample providing audio recording (n=80)	
	n[a]	(per cent)	n[b]	(per cent)
Sex				
female	183/308	(59.4)	50/78	(64.1)
male	125/308	(40.6)	28/78	(35.9)
Age				
< 20 years	3/304	(1.0)	-	-
20 to 29 years	13/304	(4.2)	3/78	(3.8)
30 to 39 years	42/304	(13.6)	10/78	(12.8)
40 to 49 years	62/304	(20.1)	16/78	(20.5)
50 to 59 years	65/304	(21.1)	16/78	(20.5)
60 to 69 years	69/304	(22.4)	20/78	(25.6)
70 to 79 years	42/304	(13.6)	11/78	(14.1)
> 79 years	12/304	(3.9)	2/78	(2.6)
Family status				
never married	71/299	(23.7)	21/74	(28.4)
married	156/299	(52.2)	34/74	(45.9)
divorced	47/299	(15.7)	13/74	(17.6)
widowed	25/299	(8.4)	6/74	(8.1)
Formal education				
low	130/304	(42.8)	40/77	(51.9)
medium	106/304	(34.9)	27/77	(35.1)
high	68/304	(22.4)	10/77	(13.0)
Mother tongue				
German	279/303	(92.1)	75/76	(98.7)
other	24/303	(7.9)	1/76	(1.3)
Occupation				
employed	144/302	(47.7)	33/75	(44.0)
retired	110/302	(36.4)	28/75	(37.3)
homemaker	12/302	(4.0)	4/75	(5.3)
student	11/302	(3.6)	2/75	(2.7)
unemployed	23/302	(7.6)	8/75	(10.7)
other	2/302	(0.7)	-	-
Health problem consulted				
type 2 diabetes	112/308	(36.4)	31/78	(39.7)
chronic back pain	101/308	(32.8)	23/78	(29.5)
depressive disorder	83/308	(26.8)	21/78	(26.9)
other	12/308	(3.9)	3/78	(3.8)

[a] valid sample size varies between 299 and 308 due to missing values
[b] valid sample size varies between 74 and 78 due to missing values

Supplementary Table 4. Multilevel variance decomposition and association analyses of the observer measures in available cases

	Measure			
	**SDM-Q-9**	**OPTION-12**	**OPTION-5**	**4 HCS**
Sample				
number of patients	301	71	79	66
number of physicians	29	22	24	22
mean patients/physician	10.38	3.23	3.29	3.00
Descriptive parameters				
overall mean	83.44<br>(81.14 to 85.63)	16.15<br>(13.19 to 19.15)	11.83<br>(8.01 to 15.65)	33.16<br>(30.05 to 36.45)
overall variance	326.69<br>(279.60 to 385.79)	68.71<br>(45.00 to 111.00)	153.09<br>(105.70 to 228.11)	109.12<br>(73.49 to 167.61)
patient-level variance	320.36<br>(271.90 to 376.20)	30.69<br>(20.68 to 45.42)	88.94<br>(59.93 to 133.71)	77.40<br>(47.21 to 123.22)
physician-level variance	6.33<br>(0.00 to 34.14)	38.03<br>(14.87 to 78.97)	64.14<br>(16.41 to 134.51)	31.71<br>(0.01 to 93.97)
intracluster correlation	0.019<br>(0.000 to 0.101)	0.535<br>(0.288 to 0.743)	0.405<br>(0.126 to 0.639)	0.274<br>(0.000 to 0.602)
Physician-level correlations				
SDM-Q-9	-	0.12<br>(-0.00 to 0.23)	0.24<br>(0.11 to 0.37)	0.17<br>(0.04 to 0.29)
OPTION-12	-	-	0.75<br>(0.44 to 1.00)	0.69<br>(0.40 to 1.00)
OPTION-5	-	-	-	0.50<br>(0.27 to 0.75)

The estimated parameters reported with 95% credible interval.

Supplementary Table 5. Multilevel variance decomposition and association analyses of the observer measures in complete cases

	Measure			
	**SDM-Q-9**	**OPTION-12**	**OPTION-5**	**4 HCS**
Sample				
number of patients	60	60	60	60
number of physicians	21	21	21	21
mean patients/physician	2.86	2.86	2.86	2.86
Descriptive parameters				
overall mean	86.98<br>(82.94 to 91.19)	16.18<br>(12.83 to 19.46)	12.72<br>(7.77 to 17.62)	33.59<br>(30.00 to 37.46)
overall variance	274.97<br>(191.90 to 396.92)	79.06<br>(48.19 to 135.20)	158.40<br>(97.47 to 272.60)	120.97<br>(79.32 to 195.50)
patient-level variance	272.42<br>(189.80 to 394.50)	28.70<br>(18.14 to 45.70)	55.78<br>(35.61 to 87.20)	75.31<br>(44.37 to 128.71)
physician-level variance	2.55<br>(0.00 to 22.05)	50.36<br>(20.28 to 106.60)	102.62<br>(40.32 to 219.91)	45.66<br>(0.03 to 120.51)
intracluster correlation	0.009<br>(0.000 to 0.078)	0.616<br>(0.366 to 0.816)	0.626<br>(0.363 to 0.824)	0.357<br>(0.000 to 0.666)
Patient-level correlations				
SDM-Q-9	-	0.07<br>(0.03 to 0.11)	0.09<br>(0.05 to 0.14)	0.28<br>(0.20 to 0.36)
OPTION-12	-	-	0.30<br>(0.22 to 0.39)	0.51<br>(0.38 to 0.65)
OPTION-5	-	-	-	0.31<br>(0.23 to 0.40)
Physician-level correlations				
SDM-Q-9	-	0.22<br>(0.08 to 0.37)	0.11<br>(-0.02 to 0.23)	0.15<br>(0.03 to 0.28)
OPTION-12	-	-	0.81<br>(0.49 to 1.00)	0.66<br>(0.39 to 0.98)
OPTION-5	-		-	0.52<br>(0.30 to 0.78)
Overall correlations				
SDM-Q-9	-	0.11<br>(0.07 to 0.16)	0.09<br>(0.05 to 0.14)	0.24<br>(0.18 to 0.32)
OPTION-12	-	-	0.67<br>(0.51 to 0.86)	0.61<br>(0.47 to 0.79)
OPTION-5	-	-	-	0.46<br>(0.35 to 0.60)

The estimated parameters reported with 95% credible interval.

Supplementary Table 6. Inter-rater reliability of observer measures

	Measure		
	**OPTION-12**	**OPTION-5**	**4 HCS**
Fixed rater effect			
residuals	142.2[a]	158.4[b]	131.8[c]
deviance	947.5	1076.1	839.9
pD	27.5	62.3	56.4
DIC	975.0	1138.4	896.3
patient-level IRR (95% CrI)	0.121 (0.000 to 0.501)	0.678 (0.474 to 0.815)	0.759 (0.587 to 0.878)
physician-level IRR (95% CrI)	0.948 (0.832 to 0.999)	0.992 (0.959 to 1.000)	0.748 (0.001 to 0.979)
global IRR (95% CrI)	0.611 (0.401 to 0.789)	0.811 (0.697 to 0.891)	0.802 (0.688 to 0.888)
Random rater effect			
residuals	142.1[a]	157.8[b]	131.8[c]
deviance	884.9	1010.3	838.9
pD	57.9	83.8	57.2
DIC	942.8	1094.1	896.2
patient-level IRR (95% CrI)	0.434 (0.000 to 0.730)	0.786 (0.628 to 0.885)	0.760 (0.600 to 0.873)
physician-level IRR (95% CrI)	0.690 (0.387 to 0.962)	0.729 (0.369 to 0.984)	0.763 (0.003 to 0.977)
global IRR (95% CrI)	0.605 (0.397 to 0.774)	0.768 (0.624 to 0.870)	0.800 (0.686 to 0.887)

The estimated parameters reported with 95% credible interval.
[a] compare to 142 data points
[b] compare to 158 data points
[c] compare to 132 data points
pD, number of effective parameters; DIC, deviance information criterion; IRR, inter-rater reliability; CrI, credible interval

Supplementary Table 7. Model fit of the regressions models used to create skills networks

	Outcome[a]								
	**Skill 1**	**Skill 2**	**Skill 3**	**Skill 4**	**Skill 5**	**Skill 6**	**Skill 7**	**Skill 8**	**Skill 9**
Fixed intercept, fixed slopes									
residuals[b]	300.4	300.4	300.4	300.4	300.4	300.4	300.4	300.4	300.4
deviance	920.4	796.9	833.4	704.0	531.8	769.6	663.2	684.6	646.0
pD	10.0	10.0	10.0	10.0	10.0	10.0	10.0	10.0	10.0
DIC	930.3	806.9	843.3	714.0	541.8	779.6	673.1	694.5	655.9
Random intercept, fixed slopes									
residuals[b]	301.2	301.3	301.2	301.2	301.3	301.1	301.3	301.1	301.3
deviance	918.8	794.9	832.8	695.8	527.6	764.5	658.6	680.9	642.0
pD	12.6	13.0	12.0	16.7	14.7	14.8	14.8	14.2	14.4
DIC	931.3	807.9	844.8	712.6	542.3	779.3	673.4	695.1	656.4
Random intercept, random slopes									
residuals[b]	300.7	300.7	300.4	300.5	300.7	300.6	300.4	300.3	300.5
deviance	899.2	677.4	689.8	543.3	434.5	714.4	539.3	461.2	460.6
pD	35.1	69.3	79.5	84.1	63.3	51.3	72.4	88.0	77.0
DIC	934.3	746.7	769.3	627.5	497.8	765.7	611.7	549.2	537.6

[a] The outcomes are the items of the 9-item Shared Decision Making Questionnaire (SDM-Q-9)
[b] compare to 301 data points
pD, number of effective parameters; DIC, deviance information criterion

Supplementary Table 8. Multiple linear regression model weights to predict observer-rated measures of shared decision making competence from activation of the skills of the SDM-Q-9

	**OPTION-12**	**OPTION-5**	**4 HCS**
Intercept	16.15 (14.14 to 18.19)	12.44 (9.59 to 15.33)	33.20 (31.34 to 35.00)
Activation			
Skill 1	112.06[b] (24.08 to 196.51)	42.29 (-82.22 to 161.72)	23.71 (-53.83 to 99.04)
Skill 2	-5.05 (-71.69 to 62.69)	1.08 (-93.41 to 96.84)	10.95 (-47.98 to 70.84)
Skill 3	-17.64 (-134.41 to 100.00)	11.03 (-154.40 to 177.42)	3.54 (-99.77 to 107.60)
Skill 4	12.08 (-30.39 to 51.15)	13.19 (-46.70 to 68.71)	3.61 (-33.96 to 38.25)
Skill 5	4.08 (-71.20 to 78.71)	-37.82 (-144.51 to 67.70)	24.41 (-42.21 to 90.45)
Skill 6	61.97 (-9.97 to 133.40)	55.23 (-46.04 to 155.91)	26.58 (-36.92 to 89.69)
Skill 7	47.96 (-20.71 to 116.41)	42.62 (-54.47 to 139.31)	52.19 (-8.49 to 112.70)
Skill 8	132.58 (-9.25 to 269.70)	84.11 (-115.91 to 277.12)	76.76 (-48.70 to 198.10)
Skill 9	11.62 (-82.67 to 109.31)	-14.39 (-147.72 to 124.01)	23.30 (-60.07 to 109.71)
Residual variance	23.37 (9.75 to 51.72)	47.06 (19.67 to 104.20)	18.26 (7.62 to 40.42)
Model variance	24.61 (9.64 to 51.11)	33.89 (10.56 to 81.85)	11.78 (3.38 to 28.62)
R	0.713 (0.521 to 0.843)	0.638 (0.438 to 0.787)	0.616 (0.417 to 0.770)
R^2	0.515 (0.272 to 0.710)	0.415 (0.192 to 0.619)	0.387 (0.174 to 0.594)
adjusted R^2	0.151 (-0.274 to 0.492)	-0.024 (-0.415 to 0.333)	-0.072 (-0.446 to 0.289)
residuals[a]	22.0	22.0	22.0

All estimated parameters reported with 95% credible interval.

[a] compare to 22 data points

[b] regression slope parameter is not zero with a probability of at least 95%

Supplementary Table 9. Multiple linear regression model weights to predict observer-rated measures of shared decision making competence from outstrength of the skills of the SDM-Q-9

	OPTION-12	OPTION-5	4 HCS
Intercept	16.14<br>(14.00 to 18.34)	12.44<br>(9.83 to 15.08)	33.20<br>(31.78 to 34.63)
Outstrength			
Skill 1	-8.20<br>(-18.52 to 1.74)	-5.65<br>(-18.04 to 6.44)	-5.74<br>(-12.52 to 0.77)
Skill 2	1.71<br>(-19.38 to 23.16)	0.54<br>(-24.94 to 26.46)	-0.97<br>(-14.80 to 13.10)
Skill 3	3.40<br>(-8.22 to 14.81)	-4.18<br>(-18.22 to 9.60)	-2.79<br>(-10.42 to 4.69)
Skill 4	0.22<br>(-8.38 to 8.47)	2.66<br>(-7.73 to 12.63)	2.25<br>(-3.40 to 7.66)
Skill 5	4.54<br>(-6.09 to 14.41)	5.78<br>(-7.07 to 17.70)	-0.27<br>(-7.24 to 6.21)
Skill 6	10.78<br>(-1.04 to 23.14)	4.92<br>(-9.36 to 19.86)	13.07[b]<br>(5.31 to 21.18)
Skill 7	-11.54[b]<br>(-22.61 to -0.51)	-14.67[b]<br>(-28.04 to -1.35)	-7.35[b]<br>(-14.61 to -0.12)
Skill 8	0.27<br>(-9.78 to 10.16)	5.44<br>(-6.71to 17.39)	-3.32<br>(-9.91 to 3.18)
Skill 9	4.43<br>(-7.62 to 14.88)	8.41<br>(-4.94 to 22.24)	5.55<br>(-1.70 to 13.06)
Residual variance	26.97<br>(11.24 to 59.75)	39.37<br>(16.42 to 87.23)	11.61<br>(4.84 to 25.71)
Model variance	24.69<br>(8.69 to 55.23)	34.39<br>(12.01 to 76.22)	12.30<br>(4.57 to 25.86)
R	0.686<br>(0.494 to 0.819)	0.677<br>(0.477 to 0.814)	0.714<br>(0.528 to 0.839)
R^2	0.478<br>(0.244 to 0.670)	0.466<br>(0.227 to 0.662)	0.516<br>(0.279 to 0.705)
adjusted R^2	0.086<br>(-0.323 to 0.423)	0.066<br>(-0.352 to 0.408)	0.153<br>(-0.262 to0.483)
residuals[a]	22.0	22.0	22.0

All estimated parameters reported with 95% credible interval.

[a] compare to 22 data points

[b] regression slope parameter is not zero with a probability of at least 95%

Supplementary Table 10. Multiple linear regression model weights to predict observer-rated measures of shared decision making competence from instrength of the skills of the SDM-Q-9

	OPTION-12	OPTION-5	4 HCS
Intercept	16.16<br>(13.68 to 18.67)	12.44<br>(9.68 to 15.24)	33.20<br>(31.47 to 34.96)
Instrength			
Skill 1	39.48<br>(-44.31 to 123.01)	42.55<br>(-50.85 to 135.61)	39.02<br>(-19.77 to 97.60)
Skill 2	-1.19<br>(-13.37 to 11.43)	-1.19<br>(-14.76 to 12.89)	-2.30<br>(-10.84 to 6.55)
Skill 3	0.43<br>(-8.62 to 14.89)	-4.16<br>(-14.24 to 5.63)	2.61<br>(-3.74 to 8.76)
Skill 4	3.46<br>(-8.21 to 14.89)	5.87<br>(-7.14 to 18.62)	-2.90<br>(-11.08 to 5.12)
Skill 5	-9.52<br>(-25.76 to 7.10)	-3.68<br>(-21.78 to 14.86)	-1.50<br>(-12.88 to 10.16)
Skill 6	10.87<br>(-1.87 to 24.47)	14.25[b]<br>(0.04 to 29.41)	6.71<br>(-2.23 to 16.24)
Skill 7	-9.28<br>(-26.64 to 8.30)	-16.87<br>(-36.22 to 2.73)	-1.29<br>(-13.46 to 11.03)
Skill 8	-0.81<br>(-10.09 to 8.38)	2.09<br>(-8.26 to 12.34)	-1.74<br>(-8.25 to 4.70)
Skill 9	4.79<br>(-4.37 to 14.29)	7.39<br>(-2.83 to 17.98)	1.84<br>(-4.59 to 8.50)
Residual variance	35.54<br>(14.82 to 78.76)	44.19<br>(18.42to 97.92)	17.48<br>(7.29 to 38.72)
Model variance	24.25<br>(7.34 to 57.79)	34.28<br>(10.52 to 80.34)	12.00<br>(3.65 to 29.61)
R	0.627<br>(0.429 to 0.772)	0.654<br>(0.461 to 0.796)	0.628<br>(0.429 to 0.776)
R^2	0.401<br>(0.184 to 0.595)	0.435<br>(0.213 to 0.634)	0.403<br>(0.184 to 0.602)
adjusted R^2	-0.048<br>(-0.429 to 0.292)	0.011<br>(-0.378to 0.359)	-0.046<br>(-0.429 to 0.776)
residuals[a]	22.0	22.0	22.0

All estimated parameters reported with 95% credible interval.

[a] compare to 22 data points

[b] regression slope parameter is not zero with a probability of at least 95%

Supplementary Figure 1. Skills network plots of the participating physicians

Note: The plots are arranged according to the mean rank of the physicians regarding their SDM competence across observer-rated measures, from the top left (highest competence) to the bottom right (lowest competence).

A. Skills networks of physicians in the first 12 ranks (out of 24 with data on observed competence)

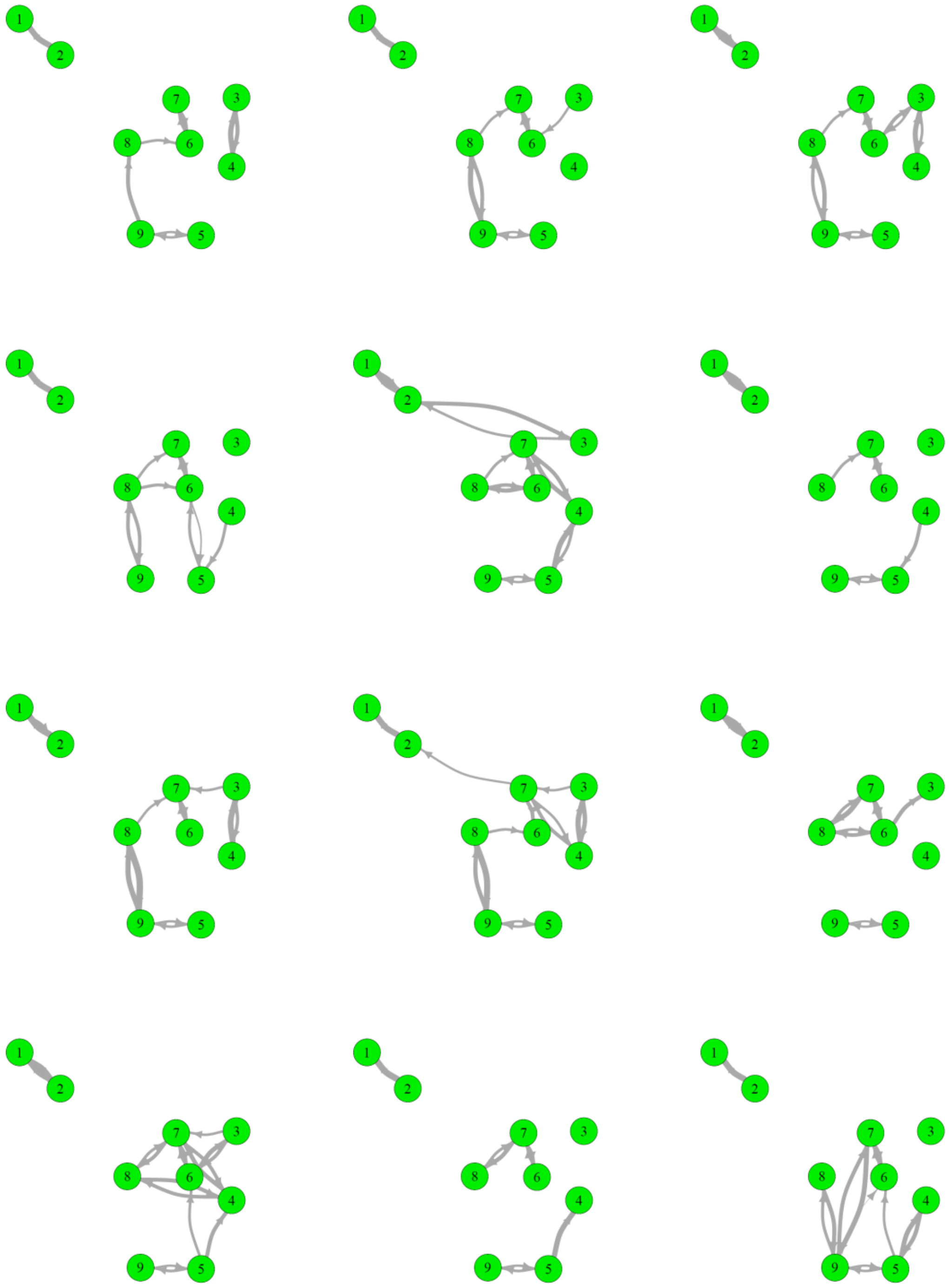

B. Skills networks of physicians in the last 12 ranks (out of 24 with data on observed competence)

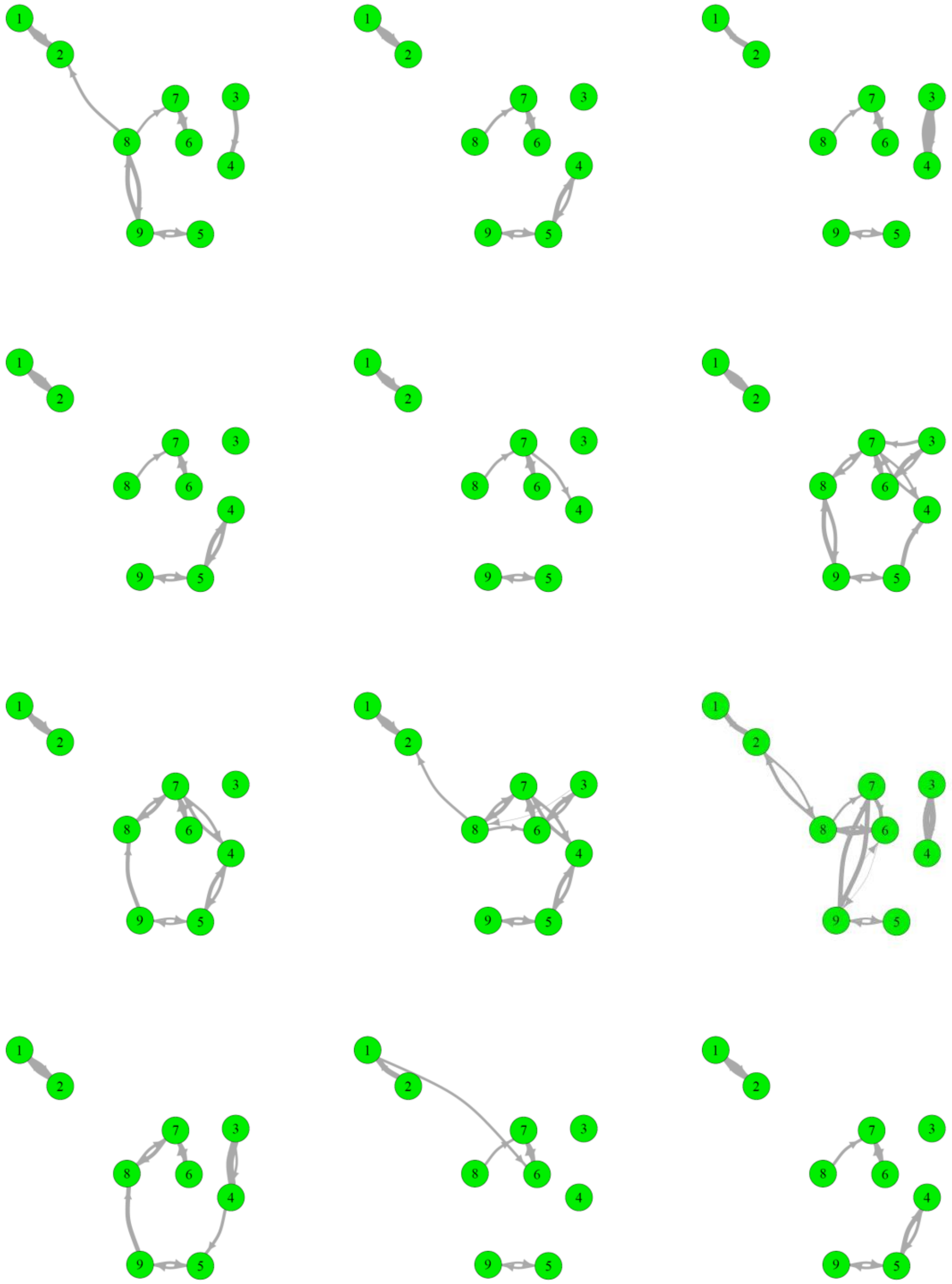

C. Skills networks of physicians without data on observed competence

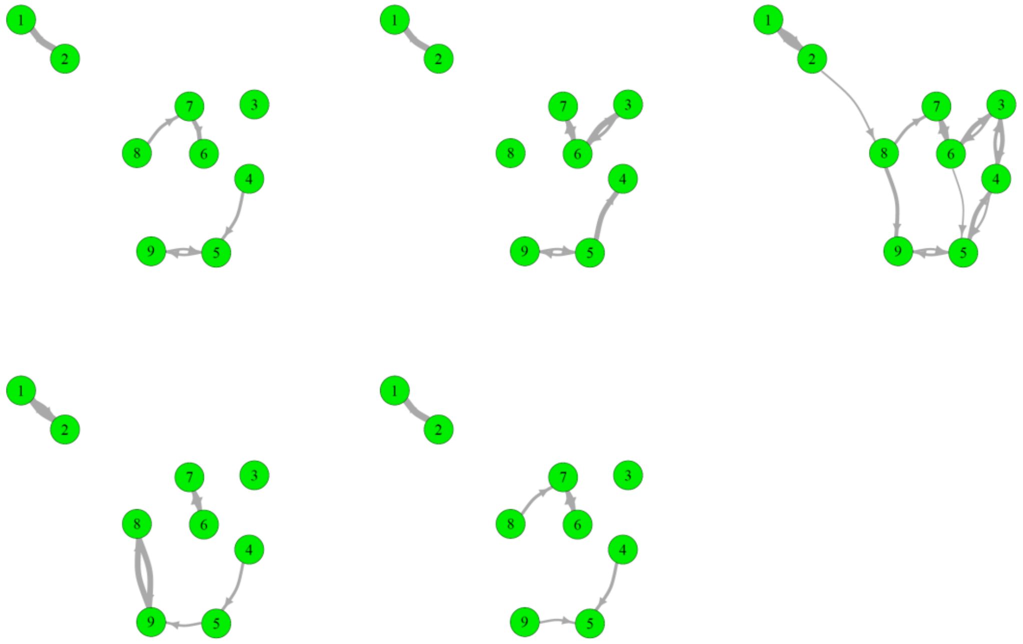

Supplement: Supplementary file 1 — Supplementary Material [file HEX-23-1466-s001.pdf]
